# Saliva Free Light Chains in Patients with Neuro-Sjögren

**DOI:** 10.3390/biomedicines10102470

**Published:** 2022-10-03

**Authors:** Franz Felix Konen, Tabea Seeliger, Philipp Schwenkenbecher, Stefan Gingele, Konstantin Fritz Jendretzky, Kurt-Wolfram Sühs, Diana Ernst, Torsten Witte, Thomas Skripuletz

**Affiliations:** 1 Department of Neurology, Hannover Medical School, Carl-Neuberg-Straße 1, 30625 Hannover, Germany; 2 Department of Immunology & Rheumatology, Hannover Medical School, Carl-Neuberg-Straße 1, 30625 Hannover, Germany

**Keywords:** Sjögren’s syndrome, Neuro-Sjögren, free light chains, KFLC, LFLC, saliva, serum, biomarker

## Abstract

**Background:** Sjögren’s syndrome (SS) is an autoimmune disease characterized by sicca symptoms and various extra-glandular manifestations. The diagnosis of SS requires sicca symptoms, anti-SSA(Ro)-antibody positivity, and/or pathological focus scores on a minor salivary gland biopsy. Previous studies have investigated different biomarkers in order to avoid invasive diagnostic procedures. It was found that kappa and lambda free light chains (KFLC and LFLC) in saliva are specific for SS. **Methods:** FLC concentrations in saliva and serum were determined in 130 patients—50 with SS and neurological involvement (Neuro-Sjögren) and 80 neurological controls. The EULAR SS disease activity index and patient reported index (ESSPRI) were determined in patients with SS. **Results:** Patients with SS revealed increased pain and decreased saliva production according to the ESSPRI and Saxon test, respectively, with increasing FLC concentrations in the saliva. No significant differences in serum and salivary protein concentrations were observed between patients with SS and controls. **Conclusion:** KFLC and LFLC concentrations in saliva are not suitable to distinguish patients with Neuro-Sjögren and neurological control subjects, thus a diagnostic biopsy is still required. The association of salivary KFLC and LFLC concentrations with saliva production and ESSPRI pain score suggests a complex relationship between dryness and pain in patients with SS.

## 1. Introduction

Sjögren´s syndrome (SS) is an autoimmune disease characterized by lymphocytic infiltration of the exocrine glands leading to sicca symptoms, but may also cause extra-glandular manifestations such as interstitial lung disease, arthritis, cutaneous vasculitis, and central or peripheral nervous system (CNS and PNS) involvement [1,2,3,4,5,6,7]. According to the latest American College of Rheumatology/European League Against Rheumatism (ACR/EULAR) classification criteria of 2016, SS can be diagnosed in a patient with sicca symptoms and additional anti-SSA(Ro)-antibody positivity and/or pathological focus scores on a minor salivary gland biopsy [8,9,10]. In addition to the established criteria for the diagnosis of SS, the search for alternative biomarkers from various body fluids continues. As B-cell hyperactivity is associated with the pathogenesis of SS and may contribute to the development of systemic manifestations, several B-cell associated biomarkers have been investigated in different body fluids, as follows: B-cell activating factor (BAFF), β2-microglobulin (β2M), soluble interleukin-2 receptor (sIL-2R), and free light chains (FLC) [11,12,13,14,15,16,17,18]. FLC are a by-product of the immunoglobulin (Ig) synthesis of B-cells and occur in a predominantly monomeric isoform (kappa free light chains (KFLC)) and a dimeric isoform (lambda free light chains (LFLC)) [13]. KFLC have already been shown to be a potential diagnostic biomarker for autoimmune-mediated diseases, particularly multiple sclerosis [19,20,21]. Increased serum KFLC and LFLC concentrations have been reported in patients with SS compared with healthy controls [14,16,17,22]. In addition, FLC concentrations have been found to be associated with disease activity according to the EULAR Sjögren’s Syndrome Disease Activity Index (ESSDAI) and the EULAR Sjögren’s Syndrome Patient-Reported Index (ESSPRI), and FLC concentrations have been proposed as biomarkers for monitoring disease activity and response to treatment [12,13,14,15,17,23,24]. As lymphocytic infiltration into exocrine glands mediates autoimmune gland inflammation, FLC concentrations in the saliva have been investigated [2,16,17]. A cut-off value for the salivary LFLC concentration of 1.1 mg/l was suggested as a possible substitute for a minor salivary gland biopsy in order to avoid invasive diagnostic procedures [16,17]. However, the transferability of these studies is limited, as they included patients with a relatively low disease activity and without neurological manifestations [16,17]. In more recent studies, the frequency of polyneuropathy in patients with SS is higher than previously described [25,26]. In a cohort of patients with SS-associated polyneuropathy (n = 44), the limbs were symmetrically affected in 84% of patients, whereas sensory function was not affected in 11% of patients, suggesting that a pure motor syndrome is also possible [25]. In this cohort of patients, electrophysiological measurements did not reveal pathognomonic findings, whereas a large proportion of patients met the diagnostic criteria of chronic inflammatory demyelinating polyneuropathy [25]. Furthermore, these patients also showed monoclonal gammopathy with monoclonal FLC [25,26,27]. Because FLC have been proposed as diagnostic and prognostic biomarkers in previous studies investigating SS patients, the role of serum and salivary FLC in patients with SS and neurological involvement needs further classification [25,26,27]. In the present study, therefore, serum and salivary protein concentrations, including KFLC and LFLC, were investigated in patients with neurological involvement of SS and in control subjects.

## 2. Materials and Methods

### 2.1. Patients

This prospective monocentric study included a total of 130 patients who presented to the Department of Neurology at Hannover Medical School (MHH) between 2019 and 2021 with symptoms or neurological signs suggestive of SS (Table 1). In 50/130 patients, the diagnosis of SS could be confirmed according to the latest classification criteria [8]. ESSPRI and ESSDAI were determined in patients with SS [23,24]. In 80/130 patients, SS could not be diagnosed because of either a negative Saxon/Schirmer test, no detection of anti-SSA(Ro)-antibodies, or a negative minor salivary gland biopsy. These patients were subsequently assigned to the control group. Here, 88% (44/50) of the SS patients suffered from neuromuscular involvement, 66% (29/44) of which suffered from immune-mediated polyneuropathies. Similarly, 86% (69/80) of the neurological control patients suffered from neuromuscular disorders, 54% (37/69) of which suffered from immune-mediated polyneuropathies. In contrast, 12% (6/50) of SS patients had involvement of the central nervous system (CNS), whereas 14% (11/80) of the control patients suffered from inflammatory CNS disorders (multiple sclerosis, immune-mediated encephalitis, or CNS vasculitis).

All of the samples were collected before treatment. Additional demographic and clinical data are shown in Table 1.

### 2.2. Saliva Sample Collection

The saliva samples were collected and processed according to the procedures described by Sandhya et al. [16,17]. Three to five milliliters of saliva were collected in the morning using the spit method, without stimulation or induction techniques. Patients were asked not to eat, drink, chew gum, or perform oral hygiene for at least 1 h before saliva collection. No special tubes or protease inhibitors were used. Saliva was processed by centrifugation at 1800× *g* for 15 min at room temperature, and was immediately frozen at −80 °C until further analytical procedures were performed.

### 2.3. Analytical Procedures

All of the serum samples were analyzed according to routine diagnostic procedures in the Neurochemistry Laboratory of the Department of Neurology at MHH. The concentrations of albumin, IgG, IgM, and IgA in the serum samples were measured by kinetic nephelometry (Beckman Coulter IMMAGE, Brea, CA, USA). The concentration of KFLC and LFLC in the saliva and serum samples were determined nephelometrically using the N Latex FLC Kappa and Lambda Kit (Siemens Healthcare Diagnostics Products GmbH, Erlangen, Germany) according to the manufacturer’s instructions, on a BN Prospec analyzer (Siemens Healthcare Diagnostics Products GmbH, Erlangen, Germany). The proposed salivary LFLC concentration cut-off of 1.1 mg/L was investigated in the present study [16].

The estimated glomerular filtration rate (eGFR) was calculated using the CKD-EPI creatinine equation [28].

Saliva production was determined using the Saxon test [8]. Here, patients chewed on a fabric gauze for 2 minutes and the weight difference before and after these 2 minutes determined the saliva production.

### 2.4. Statistical Analyses

Statistical analysis was performed using GraphPad Prism (La Jolla, CA, USA; version 5.02). The statistical significance level was set at 5%. The D’Agostino and Pearson omnibus normality test was used to assess the normal distribution of the values. Data were presented as minimum, maximum (min–max), and mean, unless otherwise stated. Fisher’s exact test was used for the group comparison of binary variables, and either the Mann–Whitney U-Test or paired t-test was used for metric variables. Longitudinal data were analyzed via Friedman test with Dunn’s multiple comparison posthoc test. Spearmans r and Pearsons r were used for a linear regression correlation analysis.

## 3. Results

### 3.1. Patients

A total of 130 patient samples from 50 patients with SS and 80 neurological controls were included in the present study (Table 1). An analysis of the demographic differences revealed a higher age for patients with SS compared with the controls (*p* = 0.0030), while all of the other demographic factors did not differ between groups. The available ESSDAI and ESSPRI scores of patients with SS are shown in Table 1.

Immunomodulatory treatment was used equally in patients with SS and controls (33/50, 66% vs. 43/80, 54%), and included the use of intravenous immunoglobulins (IVIg) (22/33, 67% vs. 30/43, 70%); prednisolone and azathioprine (6/33, 18% vs. 11/43, 26%); and cyclophosphamide, rituximab, and ocrelizumab (5/33, 15% vs. 2/43, 5%; Table 1).

### 3.2. Comparison of Patients with SS and Controls

No significant differences in serum and saliva protein concentrations were found between patients with SS and controls (Table 2). Using the proposed cut-off value for salivary LFLC concentration of 1.1 mg/L, 37/50 SS patients and 26/80 control subjects without SS were positive using this cut-off value.

### 3.3. Correlation of Serum and Saliva Protein Concentrations with Severity of Xerostomia

**Figure 1:**
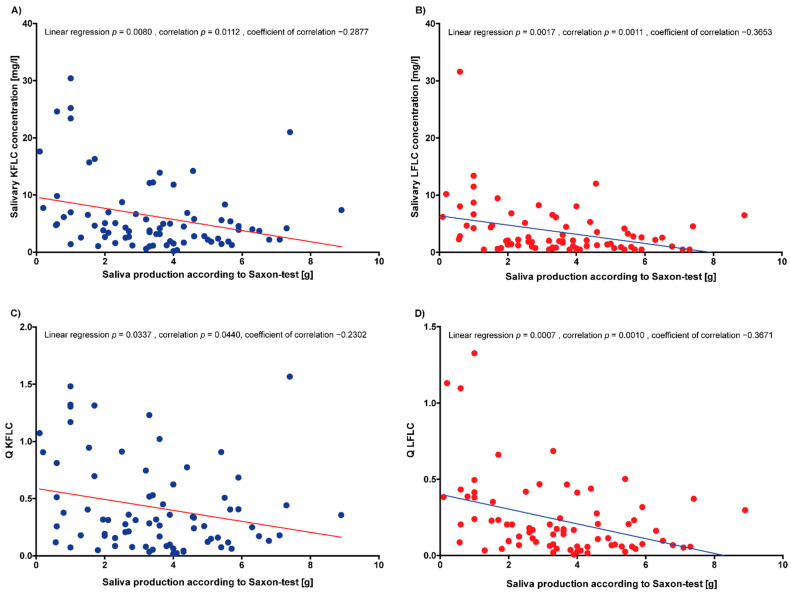
Correlations of saliva protein concentrations and quotients with saliva production. (**A**) KFLC = kappa free light chains; (**B**) LFLC = lambda free light chains; (**C)** Q KFLC = saliva/serum KFLC concentration quotient; (**D**) Q LFLC = saliva/serum LFLC concentration quotient.

Saliva production as determined by the Saxon test correlated inversely with saliva concentrations of KFLC and LFLC and saliva/serum quotients of KFLC and LFLC in all of the included patients (linear regression: *p*-values between 0.0007 and 0.0377; correlation: *p*-values between 0.0010 and 0.0440; coefficient of correlation: between −0.2302 and −0.3671; Figure 1A–D). However, the comparison of KFLC and LFLC concentrations in serum and saliva, as well as KFLC and LFLC quotients in saliva and serum between patients with and without pathological Saxon test results and patients with and without a pathological focus score on salivary minor gland biopsy did not reveal statistically significant differences (*p*-values between 0.0861 and 0.9609). Similarly, no statistically significant relationship was found in the correlation of the salivary gland biopsy focus score with the protein concentrations in the serum and saliva (*p*-values between 0.1228 and 0.9514).

### 3.4. Correlation of Serum and Saliva Protein Concentrations with Disease Activity

**Figure 2:**
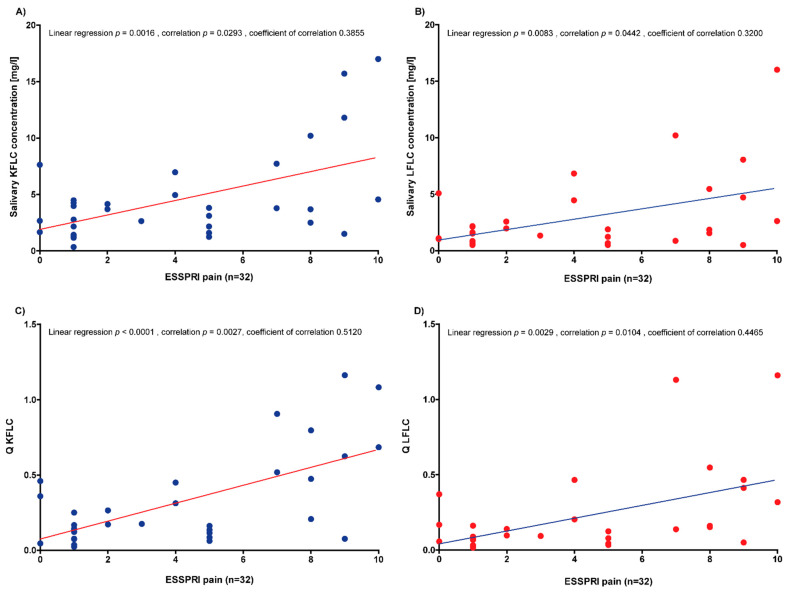
Correlation analyses of the saliva protein concentrations and quotients with the ESSPRI pain score. (**A**) KFLC = kappa free light chains; (**B**) LFLC = lambda free light chains; ESSPRI = EULAR Sjögren’s Syndrome Patient Reported Index; (**C**) Q KFLC = salivary/serum KFLC concentration quotient; (**D**) Q LFLC = salivary/serum LFLC concentration quotient.

The correlation of serum and salivary protein concentrations with the ESSDAI total score, ESSPRI total score, ESSPRI sicca score, and ESSPRI fatigue score did not reveal statistical significance (linear regression: *p*-values between 0.0513 and 0.9988; correlation: *p*-values between 0.0559 and 0.9514; Figures S1–S4, A–D). In contrast, the ESSPRI pain score was positively correlated with salivary KFLC and LFLC concentrations and salivary/serum KFLC and LFLC concentration quotients (linear regression: *p*-values between <0.0001 and 0.0083; correlation: *p*-values between 0.0027 and 0.0293; coefficient of correlation: between 0.3200 and 0.5120; Figure 2A–D).

### 3.5. Influence of IVIg Application and Longitudinal Subgroup Analysis of 20 Patients Benefitting from IVIG Treatment

A comparison of serum and saliva concentrations and quotients of the saliva and serum concentrations of KFLC and LFLC between untreated (17/50) and treated (33/50) patients with SS showed no statistically significant differences (*p*-values between 0.4425 and 0.9184).

**Figure 3:**
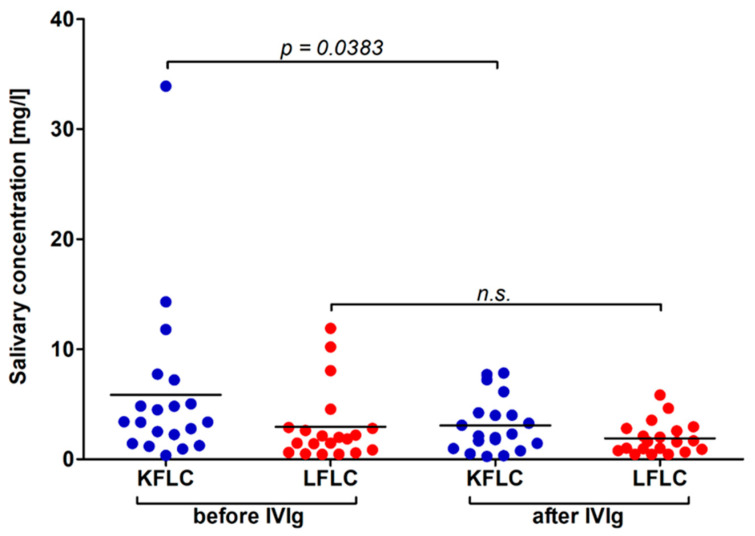
Influence of intravenous immunoglobulins on the salivary free light chain concentrations. KFLC = kappa free light chains; LFLC = lambda free light chains; IVIg = intravenous immunoglobulins; n.s. = not statistically significant.

In a total of 20 patients with immune-mediated polyneuropathies, salivary KFLC and LFLC concentrations measured immediately before and immediately after the application of IVIg. Of these patients, 14 also suffered from SS. A cumulative dose of 60–160 g IVIg was applied over 2–5 days. As all 20 patients benefited clinically from IVIg administration in terms of the stabilization of disease activity, IVIg was used as the maintenance therapy. Salivary KFLC and LFLC concentrations were lower after IVIg application, and for the KFLC concentration, this difference was statistically significant (LFLC: 2.9 mg/L vs. 1.9 mg/L; KFLC: 5.8 mg/L vs. 3.1 mg/L, *p* = 0.0383; Figure 3).

### 3.6. Correlation of Serum and Saliva FLC Concentrations

**Figure 4:**
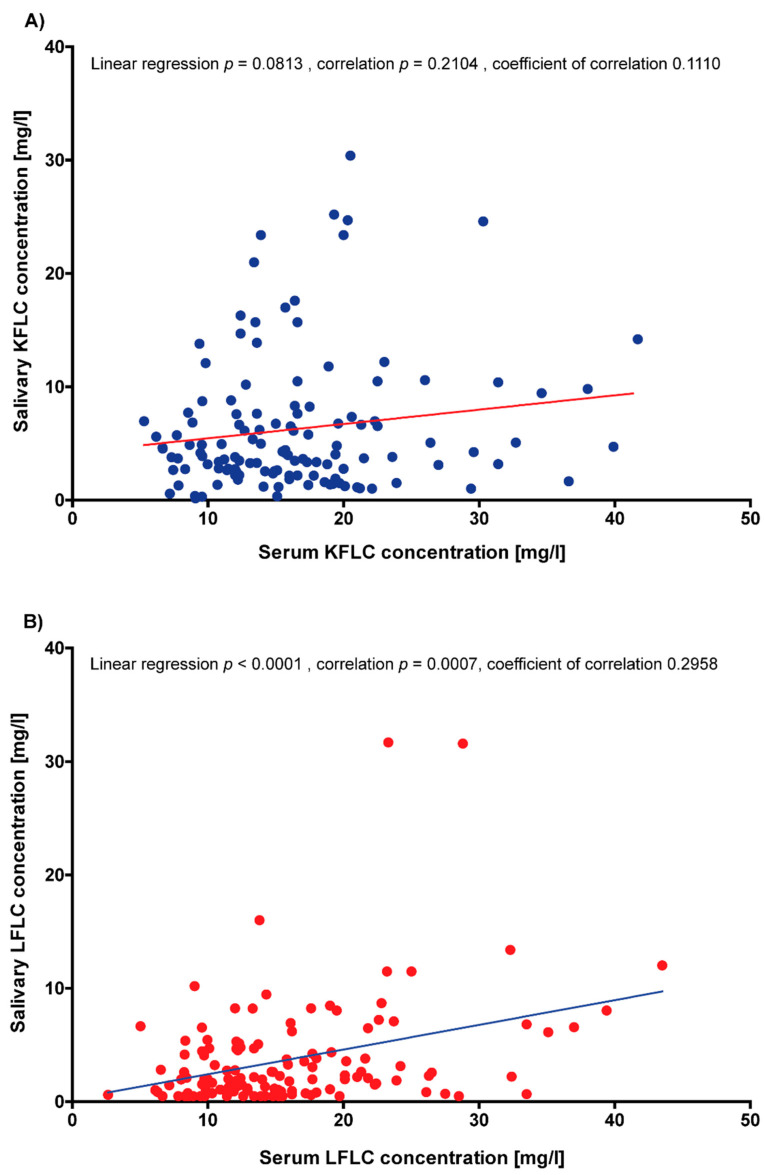
Correlation of the serum and saliva free light chain concentrations. (**A**) KFLC = kappa free light chains; (**B**) LFLC = lambda free light chains.

The correlation of KFLC concentrations in serum with KFLC concentrations in saliva did not reveal significant results (linear regression *p* = 0.0813; correlation *p* = 0.2104, coefficient of correlation 0.1110; Figure 4A). In contrast, LFLC concentrations in saliva correlated significantly with LFLC concentrations in serum (linear regression *p* < 0.0001; correlation *p* = 0.0007, coefficient of correlation 0.2958; Figure 4B).

### 3.7. Correlation of Saliva FLC Concentrations with Renal Function

No statistically significant results were found when correlating renal function estimated from eGFR according to the CKD-EPI creatinine equation in ml/min/1.73 m^2^ with salivary KFLC and LFLC concentrations (*p*-values for linear regression and a correlation between 0.1553 and 0.7927).

## 4. Discussion

The present study shows that the measurement of FLC is not useful to distinguish patients with Neuro-Sjögren from neurological control groups. First, there were no significant differences in salivary and serum FLC concentrations or in salivary and serum concentration quotients between the two groups. This finding is in contrast to other studies that investigated FLC and found significantly higher FLC concentrations in serum and/or saliva compared with controls [12,13,14,15,16,17]. Second, even the proposed cut-off concentration for salivary LFLC concentration of 1.1 mg/L, with a previously reported diagnostic sensitivity and specificity of 73% and 93%, respectively, yielded only 74% sensitivity and 33% specificity in our cohort [16]. Therefore, salivary LFLC concentration cannot be used to differentiate between Neuro-Sjögren patients and controls in our cohort either. The lack of serological and salivary group differences may be due to the fact that our study focused explicitly on patients with SS and neurological involvement. These patients have been shown to have different clinical and paraclinical features compared with patients with SS without neurological involvement [3,25]. Patients with SS-associated polyneuropathy showed pathological results not only in nerve conduction, but also in ultrasound examinations of the nerves, and often meet the diagnostic criteria for CIDP [4,25]. However, compared with patients with CIDP and without SS, a higher frequency of women and cranial nerve affection has been reported [26]. In accordance with frequent cranial nerve impairment in these patients, hearing dysfunction was found in 80% (24/30) of the patients studied [5]. Furthermore, Neuro-Sjögren patients were found to have trigeminal corneal nerve affection, which was detected by corneal confocal microscopy [29]. In addition, a significant proportion of 55% (35/64) of Neuro-Sjögren patients showed cognitive impairment in neuropsychological tests, predominantly in the form of attentional deficits [30]. Therefore, the findings of our data analysis do not necessarily contradict the previously conducted studies, but might rather further support the hypothesis that patients with SS and neurological involvement have a clinically different phenotype compared with primary rheumatic patients with SS and without neurological involvement. Another reason for the lack of differences in the present study could be the selected control patients, who were mostly diagnosed with a neurological autoimmune disease such as immune mediated polyneuropathy. As no differences in serum KFLC concentrations were found in a large study that included patients with various neurological diseases, significant differences between Neuro-Sjögren and neurological control patients are rather unlikely [31]. Therefore, it could be speculated that serum FLC, as a surrogate biomarker for B-cell mediated autoimmunity, may not detect differences between the cohorts of patients with different autoimmune disorders such as immune-mediated polyneuropathies, with and without an association to SS. This also underlines an important limitation of the present study: the lack of a healthy control group and a control group of patients with SS and without neurological involvement. Further studies examining patients with Neuro-Sjögren, SS without neurological involvement, other autoimmune mediated disorders, and healthy controls are needed in order to definitively address these issues. Furthermore, as the control patients were also significantly younger than the Neuro-Sjögren patients, it remains speculative whether some of the control patients will be diagnosed with SS in the future, which would explain the lack of differences. Despite the significant age difference, an age-related influence on FLC concentrations is unlikely, as a recent study has shown that impaired renal function is the most important factor influencing serum FLC concentrations [32]. In the present study, renal function was not statistically different between the Neuro-Sjögren patients and neurological controls.

However, in the present study, salivary KFLC and LFLC concentrations correlated negatively with saliva production, as measured by the Saxon test. Similarly, Sandhya et al. reported a positive correlation of salivary KFLC with features of dry mouth, but beyond that, there were no other statistically significant correlations of salivary FLC with sicca symptoms [17]. In addition, several studies found a weak correlation between objective and subjective indices of ocular dryness [33,34]. On the one hand, it could be hypothesized that the lower salivary fluid production in patients with SS leads to more concentrated saliva, and thus to increased FLC concentrations. On the other hand, it could be speculated that a more pronounced impairment of excretory gland function, as shown by the lower saliva production, reflects a higher inflammatory activity, which thus leads to a higher excretion of FLC into the saliva.

In the present study, salivary protein concentrations significantly correlated with the ESSPRI pain subscore. A correlation between increasing ESSPRI scores and a decrease in non-stimulated total salivary flow has been reported in the literature [34]. Interestingly, ESSPRI dryness did not correlated with tear or salivary flow, but ESSPRI pain did [35]. Therefore, the authors noted that the ESSPRI dryness score may not reflect the true assessment of dryness [35]. In contrast, a more complex relationship and a more general concept of dryness under the influence of pain and fatigue should be assumed [34,35]. Other studies investigating FLC in patients with SS reported significant correlations with the ESSDAI total score, as well as specific domains of the score with serum IgG, KFLC, and LFLC, but none of the salivary biomarkers [12,13,14,15,16,17]. The lack of such correlations could be due to the studied patient collective of Neuro-Sjögren patients or the influence of immunomodulatory treatment. Verstappen et al. reported significantly lowered serum FLC levels after the use of rituximab or abatacept, while Sandhya et al. reported lower salivary FLC concentrations in patients treated with glucocorticoids and other immunomodulatory anti-rheumatic drugs [15,17]. As it was not possible in the present study to distinguish between patients with Neuro-Sjögren and neurological controls on the basis of salivary FLC, we investigated whether salivary FLC would be suitable for monitoring immunomodulatory treatment, as suggested in the literature. When comparing untreated and treated patients, no significant differences were found between serum or salivary FLC concentrations. When considering the lack of differences between treated and untreated patients, it should be considered that most of our treated patients used intravenous therapies with treatment-free intervals such as IVIg. In all of the treated patients, the samples were obtained immediately before the infusions, so that a minimal influence of the immunomodulatory therapies was assumed. When FLC concentrations in saliva were compared immediately before and immediately after IVIg application in patients who clinically benefitted from this treatment, significantly lower KFLC concentrations became apparent. This is remarkable, as an increase in serum KFLC concentrations has been reported after treatment with IVIg [36]. A possible explanation for these rather contrary results with increased serum and decreased salivary KFLC concentrations could be the lack of correlation between the serum and salivary KFLC concentrations in the present study. The correlation of serum and saliva KFLC concentrations in the present study showed a trend towards a positive correlation of increasing saliva KFLC concentrations with increasing serum KFLC concentrations, although no statistical significance was found.

## 5. Conclusions

Salivary FLC concentrations are not suitable to distinguish patients with Neuro-Sjögren from neurological controls. Salivary FLC concentrations are related to saliva production and ESSPRI pain, suggesting a complex relationship between dryness and pain in patients with SS.

## Figures and Tables

**Table 1:** Demographic and clinical data.

Characteristic	Sjögren’s Syndrome (n = 50)	Control Group (n = 80)	*p*-Value
Age [years], median (min–max)	60 (40–84)	54 (18–82)	0.0030
Female/male—ratio	0.5	0.9	0.1473
*Clinical features*			
Objectifiable ocular sicca-syptoms, n (%)	38 (76%)	24 (30%)	<0.0001
Objectifiable oral sicca-syptoms, n (%)	30 (60%)	30 (38%)	0.0184
Pathological focus score in minor salivary gland biopsy, n (%)	34 (68%)	0	<0.0001
Ro-antibody positivity, n (%)	26 (52%)	0	<0.0001
La-antibody positivity, n (%)	3 (6%)	0	0.1583
Rheumatoid factor positivity, n (%)	7 (14%)	4 (5%)	0.1045
ESSDAI total score, median (min–max)	17 (0–42)	ND	ND
ESSPRI total score, median (min–max) *	4.5 (0–9)	ND	ND
ESSPRI sicca score, median (min–max) *	4 (1–9)	ND	ND
ESSPRI fatigue score, median (min–max) *	6.5 (0–10)	ND	ND
ESSPRI pain score, median (min–max) *	4 (0–10)	ND	ND
*Treatment*			
Untreated patients, n (%)	17 (34%)	37 (46%)	0.5779
Treated with IVIg, n (%)	22 (44%)	30 (38%)	0.8074
Treated with oral azathioprine or prednisolon, n (%)	6 (12%)	11 (14%)	0.5809
Treated with cyclophosphamide, rituximab or ocrelizumab, n (%)	5 (10%)	2 (2%)	0.2285

ESSPRI = EULAR Sjögren’s Syndrome Patient Reported Index; ESSDAI = EULAR Sjögren’s syndrome disease activity index; IVIg = intravenous immunoglobulins; * ESSPRI scores available in 32/50 patients with SS.

**Table 2:** Serum and saliva analyses.

Characteristic	Sjögren’s Syndrome (n = 50)	Control Group (n = 80)	*p*-Value
Serum albumin concentration [g/L], mean (min–max)	40 (27–58)	42 (31–51)	0.1262
Serum IgG concentration [g/L], mean (min–max)	13 (0.4–49)	12 (4–32)	0.8991
Serum IgA concentration [g/L], mean (min–max)	2 (0.1–5.1)	2 (0.5–8.8)	0.6153
Serum IgM concentration [g/L], mean (min–max)	1.2 (0.03–7.7)	1.2 (0.28–9.6)	0.1538
Serum KFLC concentration [mg/L], mean (min–max)	19 (5–124)	17 (7–42)	0.4023
Serum LFLC concentration [mg/L], mean (min–max)	14 (3–131)	15 (7–172)	0.8802
Salivary KFLC concentration [mg/L], mean (min–max)	7 (0.4–30)	7 (0.2–60)	0.8183
Salivary LFLC concentration [mg/L], mean (min–max)	4 (0.5–32)	3 (0.5–32)	0.4934
Saliva/serum KFLC quotient, mean (min–max)	0.5 (0.02–1.7)	0.4 (0.02–2.4)	0.8764
Saliva/serum LFLC quotient, mean (min–max)	0.3 (0.02–1.3)	0.2 (0.01–1.4)	0.3983
eGFR [mL/min/1.73 m^2^], mean (min–max)	82 (51–118)	87 (39–134)	0.1372

Ig = immunoglobulin; KFLC = kappa free light chain; LFLC = lambda free light chain; eGFR = estimated glomerular filtration rate according to the CKD-EPI creatinine equation.

## Data Availability

The datasets used and/or analyzed during the current study areavailable from the corresponding author upon reasonable request.

## Supplementary Materials

The following supporting information can be downloaded at: https://www.mdpi.com/article/10.3390/biomedicines10102470/s1, Figure S1: Correlations of saliva protein concentrations and quotients with ESSDAI total score. ESSDAI = EULAR Sjögren’s syndrome disease activity index; KFLC = kappa free light chains; LFLC = lambda free light chains; Q KFLC = saliva/serum KFLC concentration quotient; Q LFLC = saliva/serum LFLC concentration quotient. Linear regressions as well as correlations were not statistically significant. Figure S2: Correlations of saliva protein concentrations and quotients with ESSPRI total score. ESSPRI = EULAR Sjögren’s Syndrome Patient Reported Index; KFLC = kappa free light chains; LFLC = lambda free light chains; Q KFLC = saliva/serum KFLC concentration quotient; Q LFLC = saliva/serum LFLC concentration quotient. Linear regressions as well as correlations were not statistically significant. Figure S3: Correlations of saliva protein concentrations and quotients with ESSPRI fatigue score. ESSPRI = EULAR Sjögren’s Syndrome Patient Reported Index; KFLC = kappa free light chains; LFLC = lambda free light chains; Q KFLC = saliva/serum KFLC concentration quotient; Q LFLC = saliva/serum LFLC concentration quotient. Linear regressions as well as correlations were not statistically significant. Figure S4: Correlations of saliva protein concentrations and quotients with ESSPRI sicca score. ESSPRI = EULAR Sjögren’s Syndrome Patient Reported Index; KFLC = kappa free light chains; LFLC = lambda free light chains; Q KFLC = saliva/serum KFLC concentration quotient; Q LFLC = saliva/serum LFLC concentration quotient. Linear regressions as well as correlations were not statistically significant.
