# Supplementary material for: Saliva Free Light Chains in Patients with Neuro-Sjögren
Source: Biomedicines. 2022 Oct 3;10(10):2470. doi: 10.3390/biomedicines10102470 (PMC9599066; doi:10.3390/biomedicines10102470)
Supplement: Supplementary file 1 [file biomedicines-10-02470-s001.zip › Supplemental figure legend.pdf]

Supplemental Figure S1. Correlations of saliva protein concentrations and quotients with ESSDAI total score.

ESSDAI = EULAR Sjögren's syndrome disease activity index, KFLC = kappa free light chains, LFLC = lambda free light chains, Q KFLC = saliva/serum KFLC concentration quotient, Q LFLC = saliva/serum LFLC concentration quotient. Linear regressions as well as correlations were not statistically significant.

Supplemental Figure S2. Correlations of saliva protein concentrations and quotients with ESSPRI total score.

ESSPRI = EULAR Sjögren's syndrome Patient Reported Index, KFLC = kappa free light chains, LFLC = lambda free light chains, Q KFLC = saliva/serum KFLC concentration quotient, Q LFLC = saliva/serum LFLC concentration quotient. Linear regressions as well as correlations were not statistically significant.

Supplemental Figure S3. Correlations of saliva protein concentrations and quotients with ESSPRI fatigue score.

ESSPRI = EULAR Sjögren's syndrome Patient Reported Index, KFLC = kappa free light chains, LFLC = lambda free light chains, Q KFLC = saliva/serum KFLC concentration quotient, Q LFLC = saliva/serum LFLC concentration quotient. Linear regressions as well as correlations were not statistically significant.

Supplemental Figure S4. Correlations of saliva protein concentrations and quotients with ESSPRI sicca score.

ESSPRI = EULAR Sjögren's syndrome Patient Reported Index, KFLC = kappa free light chains, LFLC = lambda free light chains, Q KFLC = saliva/serum KFLC concentration quotient, Q LFLC = saliva/serum LFLC concentration quotient. Linear regressions as well as correlations were not statistically significant.
