# Supplementary figures and images for: Saliva Free Light Chains in Patients with Neuro-Sjögren
Source: Biomedicines. 2022 Oct 3;10(10):2470. doi: 10.3390/biomedicines10102470 (PMC9599066; doi:10.3390/biomedicines10102470)

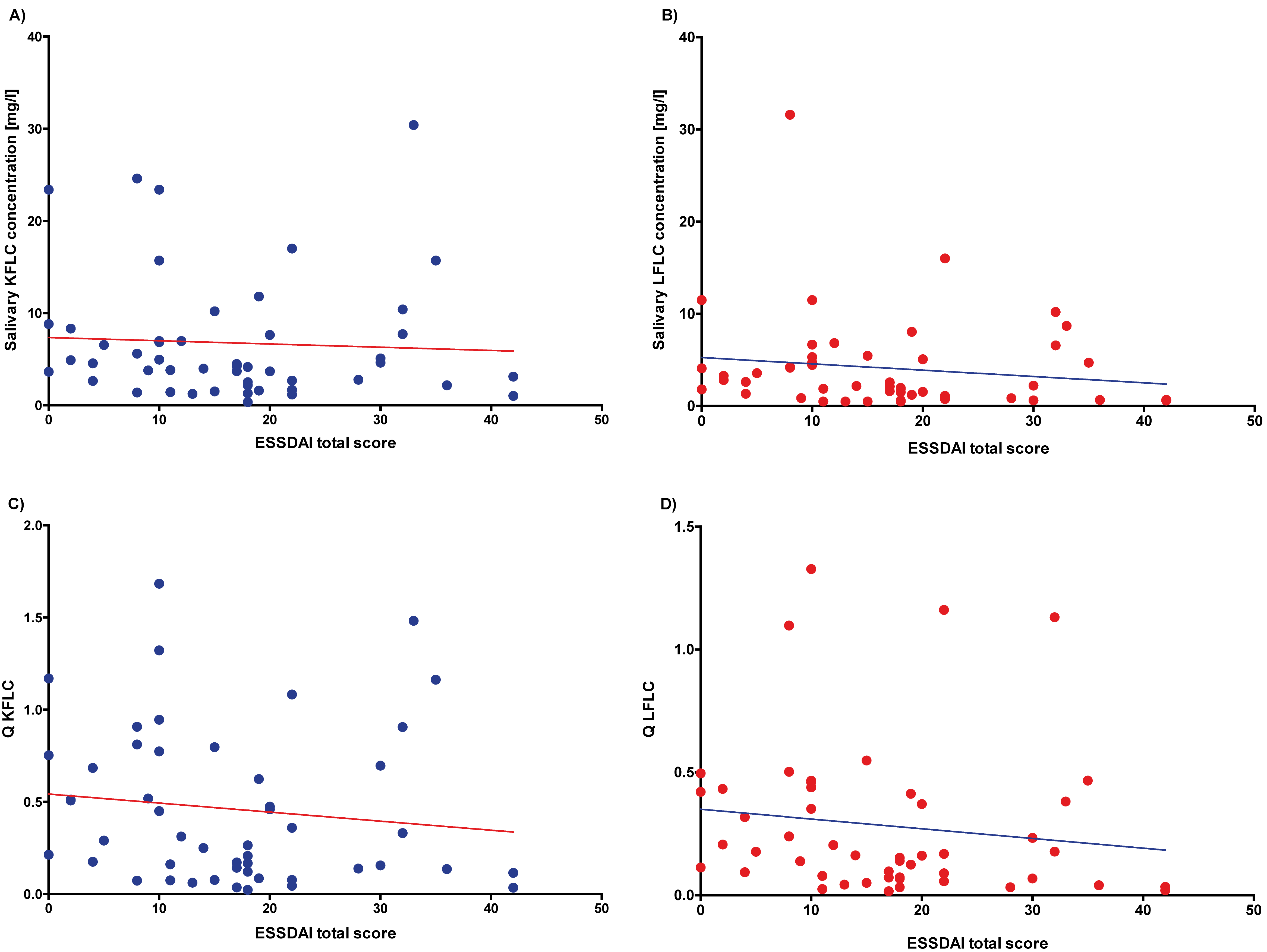

Supplement: Supplementary file 1 [file biomedicines-10-02470-s001.zip › Supplemental Figure S1.tif]

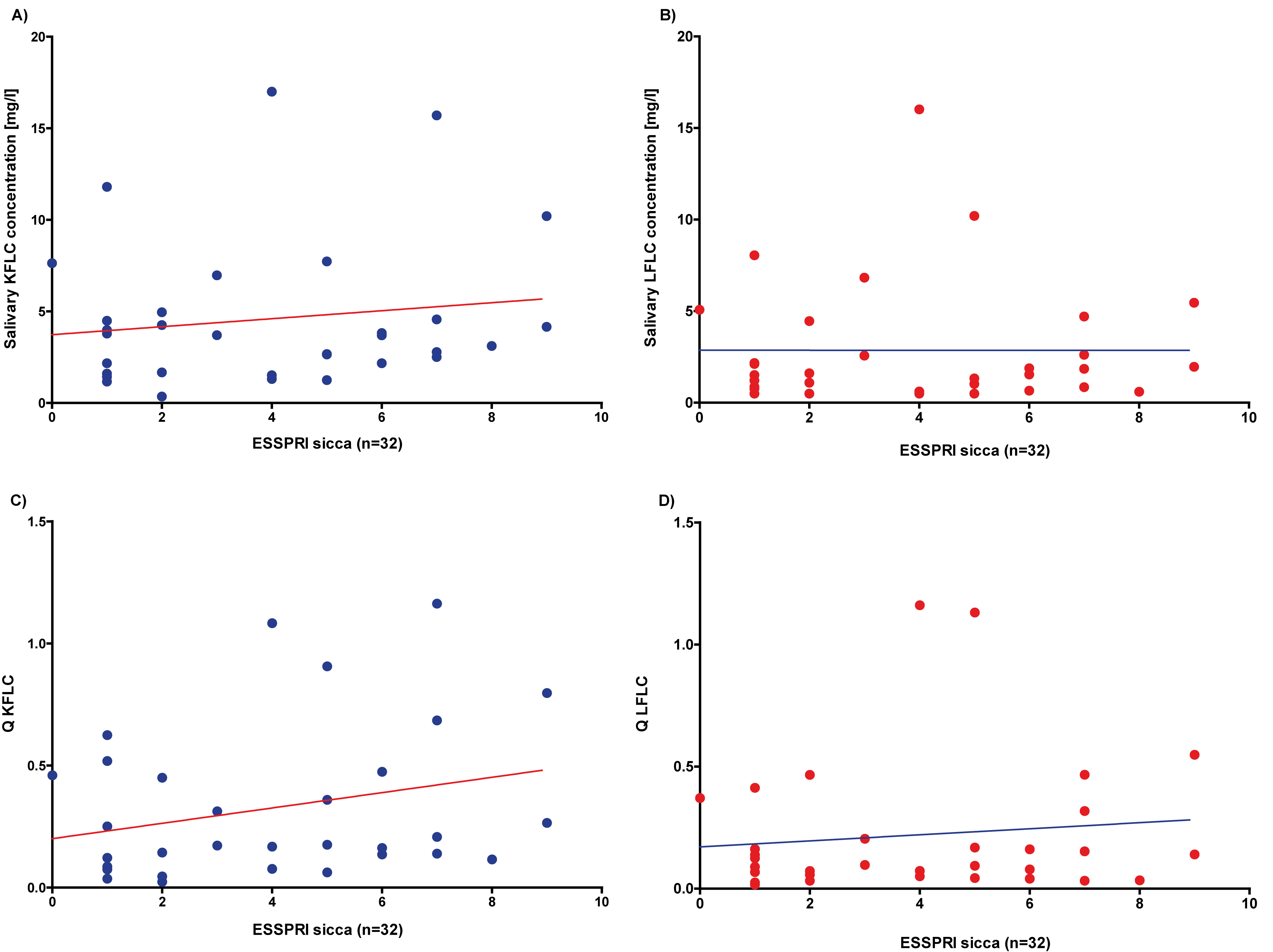

Supplement: Supplementary file 1 [file biomedicines-10-02470-s001.zip › Supplemental Figure S2.tif]

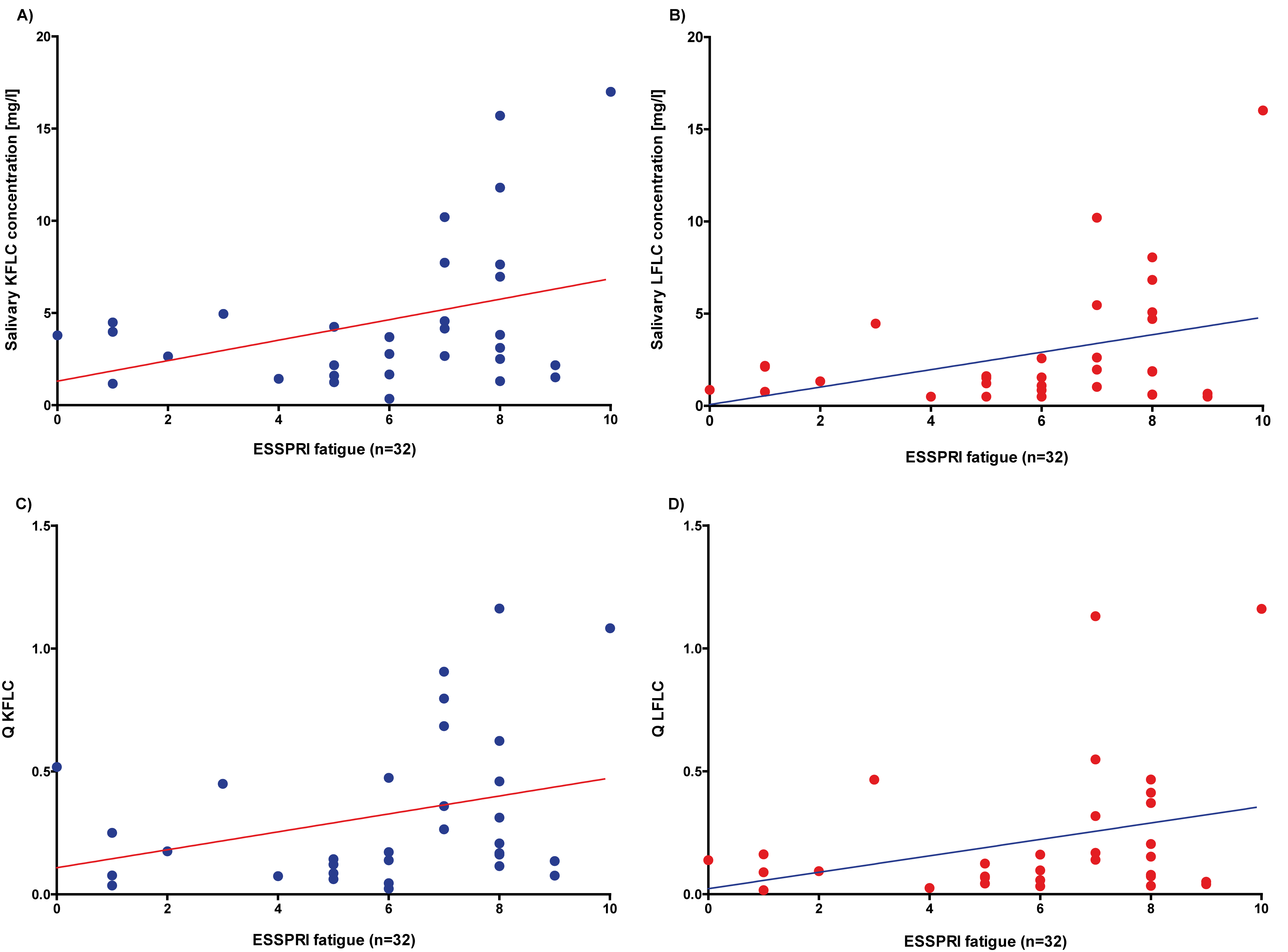

Supplement: Supplementary file 1 [file biomedicines-10-02470-s001.zip › Supplemental Figure S3.tif]

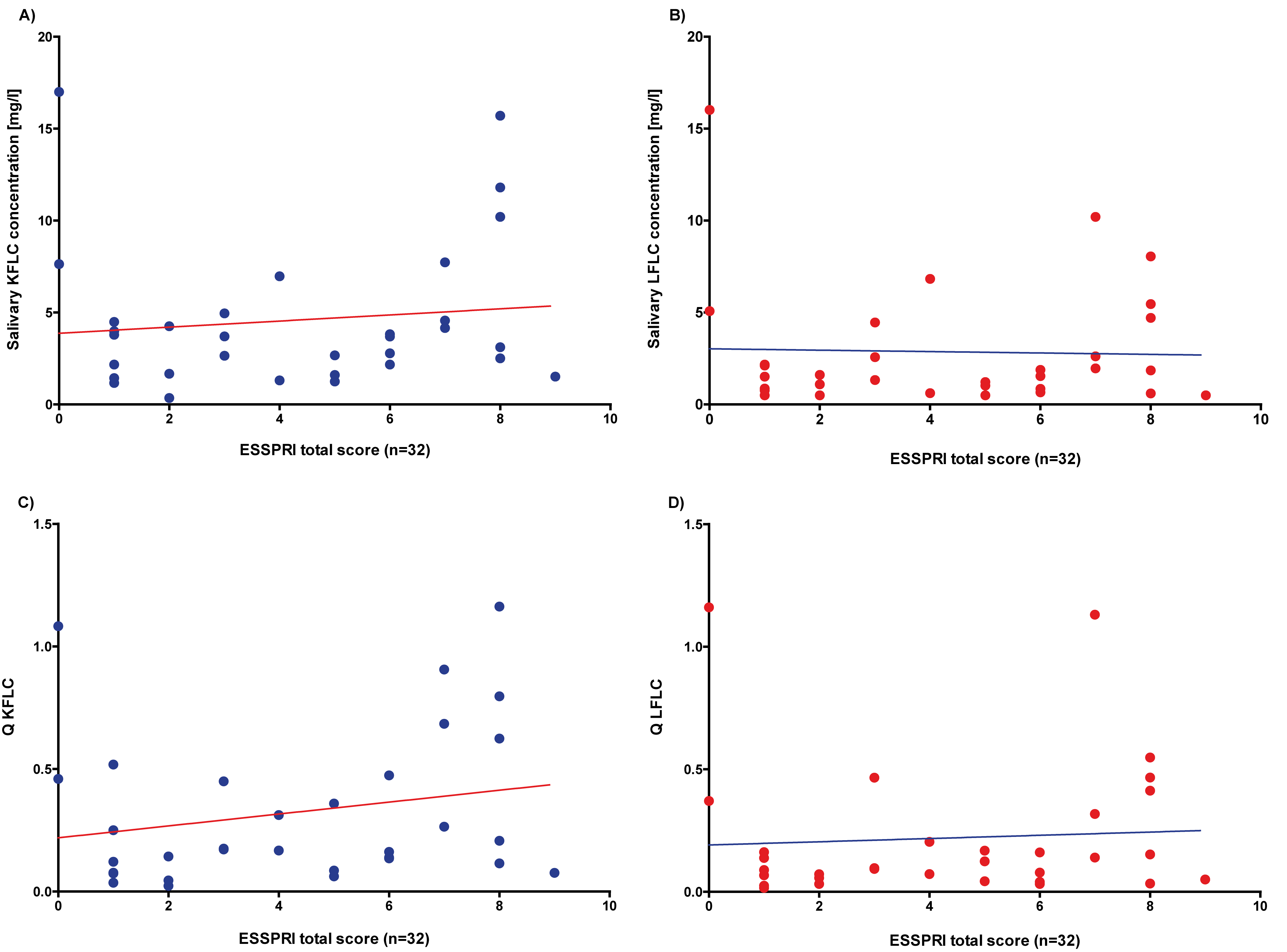

Supplement: Supplementary file 1 [file biomedicines-10-02470-s001.zip › Supplemental Figure S4.tif]
